# Phenomena Occurring upon the Sintering of a Mixture of Yttria–Zirconia Nanometric Powder and Sub-Micrometric Pure Zirconia Powder

**DOI:** 10.3390/ma14226937

**Published:** 2021-11-16

**Authors:** Kamil Wojteczko, Zbigniew Pędzich, Dariusz Zientara, Katarzyna Berent, Krzysztof Haberko

**Affiliations:** 1 Faculty of Materials Science and Ceramics, AGH University of Science and Technology, 30-059 Cracow, Poland; pedzich@agh.edu.pl (Z.P.); zientara@agh.edu.pl (D.Z.); haberko@agh.edu.pl (K.H.); 2 Academic Centre for Materials and Nanotechnology, AGH University of Science and Technology, 30-059 Cracow, Poland; kberent@agh.edu.pl

**Keywords:** ZrO_2_-Y_2_O_3_ hydrothermal crystallization, sintering, microstructure, matter transfer

## Abstract

Mixtures of powders essentially differing in their particle morphology and size were applied to prepare polycrystals in a Y_2_O_3_-ZrO_2_ system. An yttria–zirconia solid solution nanometric powder with a Y_2_O_3_ concentration of 3.5% was prepared by subjecting co-precipitated gels to hydrothermal treatment at 240 °C. The crystallization occurred in distilled water. The pure zirconia powders composed of elongated and sub-micrometer size particles were also manufactured through the hydrothermal treatment of pure zirconia gel, although in this case, the process took place in the NaOH solution. Mixtures of the two kinds of powder were prepared so as to produce a mean composition corresponding to an yttria concentration of 3 mol%. Compacts of this powder mixture were sintered, and changes in phase composition vs. temperature were studied using X-ray diffraction. The dilatometry measurements revealed the behavior of the powder compact during sintering. The polished surfaces revealed the microstructure of the resulting polycrystal. Additionally, the electron back scattering diffraction technique (EBSD) allowed us to identify symmetry between the observed grains. Hardness, fracture toughness, and mechanical strength measurements were also performed.

## 1. Introduction

For 40 years, tetragonal zirconia polycrystals composed of the yttria solid solution in zirconia (TZP) have been the subject of numerous investigations [1,2,3]. This is due to their good mechanical properties, especially their high fracture toughness. If the grain sizes of these polycrystals are sufficiently small, their grains of tetragonal symmetry can be retained in the final material. The most commonly applied yttria–zirconia solid solution usually contains 3 mol% Y_2_O_3_. The reason for the high fracture toughness of this material is related to the martensitic transformation of the tetragonal symmetry grains to their monoclinic form (t→m) at the crack tip advancing through the material. This consumes the transformation strain energy that would otherwise propagate the crack.

Grain growth usually occurs during the heat treatment of ceramic polycrystals. The driving force of this process is related to the curvature of the grain boundaries. Diffusion-controlled grain boundary migration (DIGM) and chemically controlled grain boundary migration (CIGM) were observed in studies on the behavior of Y_2_O_3_-ZrO_2_ polycrystals [4,5].

The research work was of a cognitive nature. In the classical powders systems, the phenomena observed by our team did not occur.

A desirable application in the future is the directional toughening of zirconia systems only by the change of the microstructure caused by the elongated zirconia particles.

The aim of this particular work was to show/indicate origins of bigger grains and phenomena leading to their creation. The present investigation was focused on the phase and microstructure behavior of a mixture of a 3.5 mol% Y_2_O_3_-ZrO_2_ nanometric powder and pure sub-micrometric zirconia powder compact. The size of the latter particles was one order of magnitude larger than the former.

The powders were prepared by crystallization under hydrothermal conditions, which was the subject of our previous studies [6,7]. The shape and size of the crystallites depends on the environment in which this process takes place. Isometric crystallites of up to 10 (nm) appear when the process is performed in pure water. Crystallization in strong hydroxides (NaOH, KOH, and LiOH) leads to the production of elongated particles of sub-micrometric sizes.

## 2. Materials and Methods

In the case of pure zirconia powder, the zirconium oxychloride (ZrOCl_2_) solution (2.1 M) was introduced into an ammonia solution of 3 M concentration. The resulting zirconia amorphous gel was washed with distilled water until no reaction between Cl^−^ ions and AgNO_3_ could be detected in the filtrate. Next, the gel was washed several times with a 4 M NaOH solution. The final concentration of sodium hydroxide in the filtrate corresponded to 3.8 M. The gel was then subjected to hydrothermal treatment at 240 °C for 4 h, with a rate of temperature increase of 5 °C/min. Parr equipment (type 4838) was used. Subsequently, the sodium hydroxide was removed from the powder suspension through intensive washing with distilled water.

The 3.5 mol% Y_2_O_3_-ZrO_2_ solid solution powder was also crystallized under hydrothermal conditions. In order to do this, gels with proper compositions had to be prepared by introducing the respective aqueous solutions of zirconium oxychloride and Y(NO_3_)_3_ to the vigorously stirred ammonia solution (4 M). The final pH = 9 of the system led to the quantitative precipitation of both constituents of the system. The resulting gel was then washed with water in order to remove the by-products of the process (NH_4_Cl and NH_4_NO_3_). Hydrothermal treatment, at the same conditions as those shown above, was the next step in the process. Therefore, in this case, crystallization proceeded in a pure water environment.

Suspensions of the known concentration of these powders were collected for the subsequent processing. As is shown later, the pure zirconia powder crystalized in the NaOH solution was characterized by sub-micrometric elongated particles, and one of the 3.5 mol% Y_2_O_3_-ZrO_2_ solid solutions crystallized in pure water contained isometric and nanometric particles.

The powders, i.e., the pure zirconia powder crystallized under basic conditions and the powder comprising yttria–zirconia solid solutions crystallized in pure water, were mixed. The mean composition of the mixture corresponded to a 3 mol% yttria content. The ratio between the powders corresponded to 13.94 wt% of pure zirconia and the rest of one 3.5 mol% Y_2_O_3_-ZrO_2_ solid solution powder.

A matter of utmost importance is good homogenization of the powder mixture. To achieve such a mixture, the powder suspensions were subjected to vigorous ultrasonic agitation and then, during agitation, introduced into liquid nitrogen using a peristaltic pump. The frozen mixtures were then freeze-dried using SRK System Technik (mod.GT2 Basic) equipment (SRK Systemtechnik GmbH, Riedstadt, Germany). This procedure resulted in the production of extremely soft agglomerates [8]. This helped to produce a uniform powder compaction after pressing, which resulted in good densification during sintering.

The constituent powders were characterized by their specific surface area measurements using the nitrogen adsorption (BET isotherm) and pore size distribution in the powder compact, by applying the capillary condensation method (BJH) using Micromeritics equipment (Asap 2000, Micromeritics, Norcross, GA, USA). Additionally, these powders were observed under an electron transmission microscope (FEI Tecnai FEG, 200 kV, Thermo Fisher Scientific, Hillsboro, OH, USA).

Uniaxial pressing (50 MPa), followed by cold isostatic re-pressing at 250 MPa, were applied to prepare cylindrical samples of 20 mm diameter and about 3 mm thickness. These samples were used to measure their shrinkage vs. temperature, with a rate of temperature increase of 5 °C/min up to 1400 °C. A Netzsch DIL 402C dilatometer was applied. X-ray diffraction equipment (CuKα radiation, Empyrean PANalytical, X’Pert High Score Plus v3.05, Malvern Panalytical, Malvern, UK) allowed us to determine the phase composition of the powders and sintered samples using the Rietveld method. In the case of the powders, the X-ray line broadening allowed us to assess the particle sizes according to the Scherrer formula. The sintering was also performed in the furnace with the MoSi_2_ heating elements to 1400 °C, with a rate of temperature increase of 5 °C/min and 2 h soaking time.

The SEM micrographs of the polished samples thermally etched at 1150 °C for 20 min allowed us to reveal their microstructures. We applied an FEG-SEM equipped with an Everhart–Thornley detector and an energy dispersive spectrometer (EDS). In order to identify the symmetry of the observed phases, the electron back scattering diffraction technique (EBSD) was used. The surface of the samples was ion-polished with argon ions at 3 kV for 20 min in an ion milling system (Hitachi). The EBSD measurements were performed using a FEI Versa 3D scanning electron microscope (SEM) equipped with an Oxford Instruments Symmetry C2 CMOC EBSD detector. Aztec software was used for the acquisition and post-processing.

The hardness and fracture toughness were measured using the polished but not etched samples. Future Tech (Japan) equipment was used. In the case of hardness, a load that was sufficiently low to avoid crack formation was applied. Higher loads resulting in Palmqvist cracks were used to calculate the K_Ic_ values, based on the Niihara formula [9,10]:

(1) $$K_{IC}=0.018HV^{0.6}E^{0.4}2al^{-0.5}$$

where *l* is the length of the crack and *a* is the half of the indent. Young modulus, *E* = 200 GP, was assumed.

The biaxial flexure test, i.e., the piston-on-three-ball-test (ISO 6872:2015), was used to determine the strength of the samples sintered at 1400 °C.

## 3. Results

**Figure 1:**
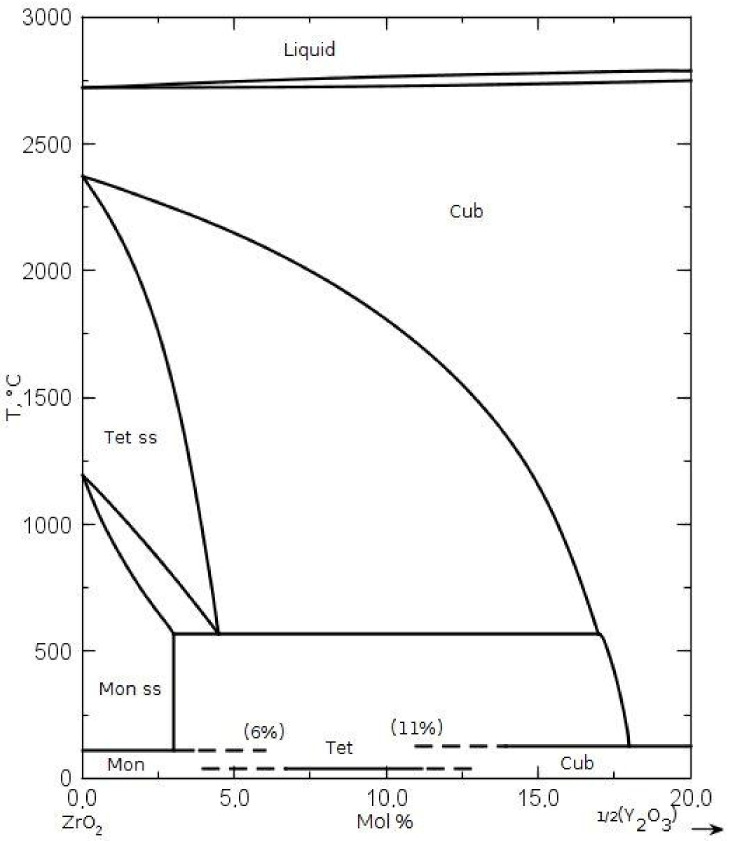
Phase diagram of the ZrO_2_-Y_2_O_3_ system [11].

The pure zirconia powder crystallized in the NaOH solution and the 3.5 mol% Y_2_O_3_-ZrO_2_ solid solution powder processed in the distilled water differed substantially in their specific surface area, phase composition, and crystallite size, as assessed on the X-ray reflections’ broadening. The data are presented in Table 1. In the case of 3.5 mol% of Y_2_O_3_-ZrO_2_ powder, we observed the existence of the phase of tetragonal symmetry, not foreseen by the phase diagram of the Y_2_O_3_-ZrO_2_ system (Figure 1). It most likely occurs due to the nanometric sizes of crystallites of this powder.

**Figure 2:**
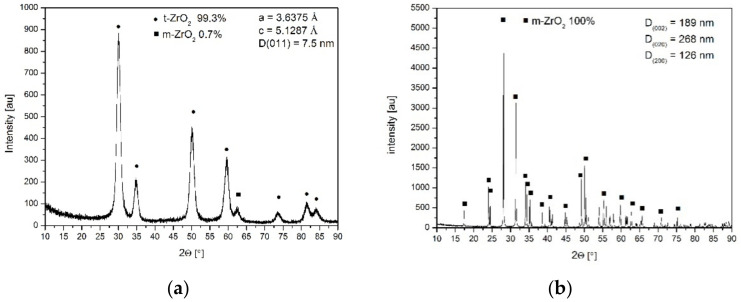
X-ray diffraction patterns of the constituent powders: (**a**) 3.5 mol% Y_2_O_3_-ZrO_2_ powder crystallized in water, in majority tetragonal symmetry phase is present; (**b**) ZrO_2_ powder crystallized in the NaOH solution, only monoclinic zirconia is present.

**Figure 3:**
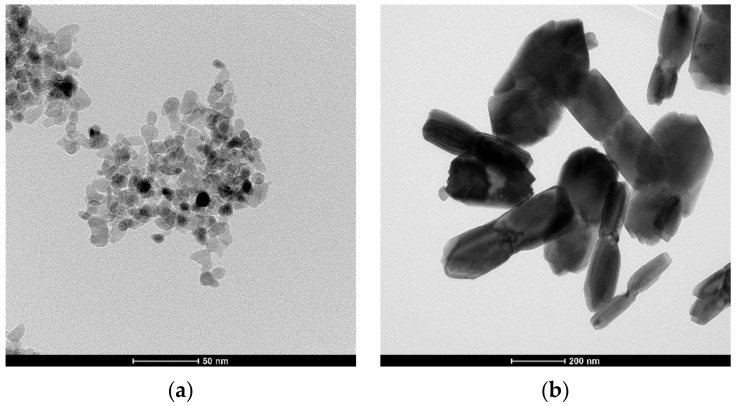
TEM micrographs of the powders crystallized under hydrothermal conditions: (**a**) Y_2_O_3_-ZrO_2_ powder crystallized under hydrothermal conditions in water; (**b**) pure zirconia powder crystallized in an NaOH solution.

Figure 2a presents the X-ray diffraction pattern of the nanometric powder crystallized in distilled water. Figure 2b shows the pattern of the pure zirconia powder crystallized in the NaOH solution. In the latter case, the monoclinic pure zirconia powder (Figure 2b) of different crystallite sizes assessed using X-ray line broadening of different reflections demonstrated an anisotropic shape. The isotropic shape of the ss powder crystallized in water and the anisotropic shape of the pure zirconia crystallized in the NaOH solution are demonstrated in the TEM micrographs (Figure 3). These observations agree with our previous studies on the effect of the crystallization environment under hydrothermal conditions on the particle shapes [6,7].

**Figure 4:**
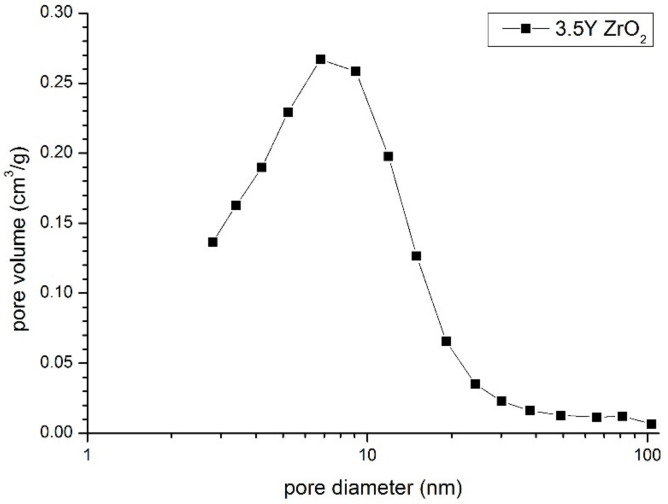
Pore size distribution curve of the powder compact of the powder mixture.

Figure 4 shows the pore size distribution in the compact of both powder mixtures. Its mono-modal shape indirectly demonstrates good powder homogenization. This was substantiated by its single-mode pore sizes (about 8 nm), which were close to the crystallite sizes from the nanometric powder crystallized in water (Table 1). The second and much higher pore modal size should be expected if sub-micrometer pure zirconia particles form isolated clusters. A lack of such pores indicates that these particles are separated by the yttria–zirconia part of the system. The same pore size distribution characteristics were also observed using Hg-porosimetric measurements.

**Figure 5:**
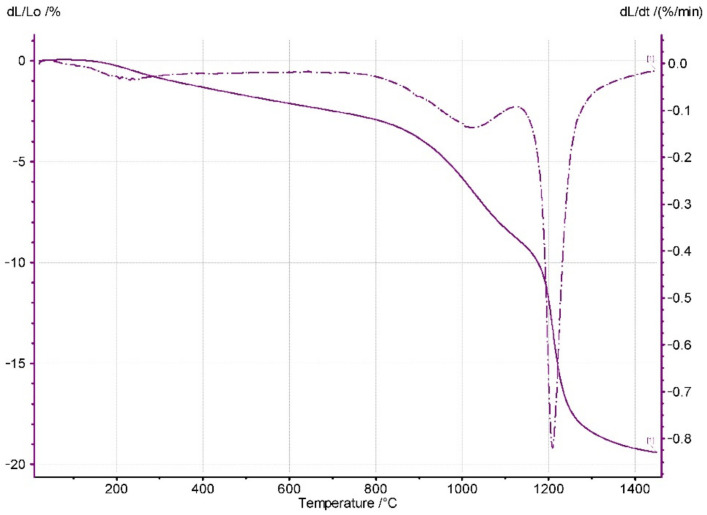
Shrinkage vs. temperature of the powder compact. Rate of the temperature increase: 5 °C/min.

Figure 5 shows the dilatometric curve of the powder compact shrinkage vs. temperature. Shrinkage starts at very low temperatures. This phenomenon is related to the desorption of water molecules from the large surface area of the nanometric particles [8]. Therefore, in the studied system, the contact points between the nanometric particles of the Y_2_O_3_-ZrO_2_ ss particles were responsible for the low temperature shrinkage. Further temperature increases led to the vast densification of the system, which was undoubtedly due to solid-state sintering.

**Figure 6:**
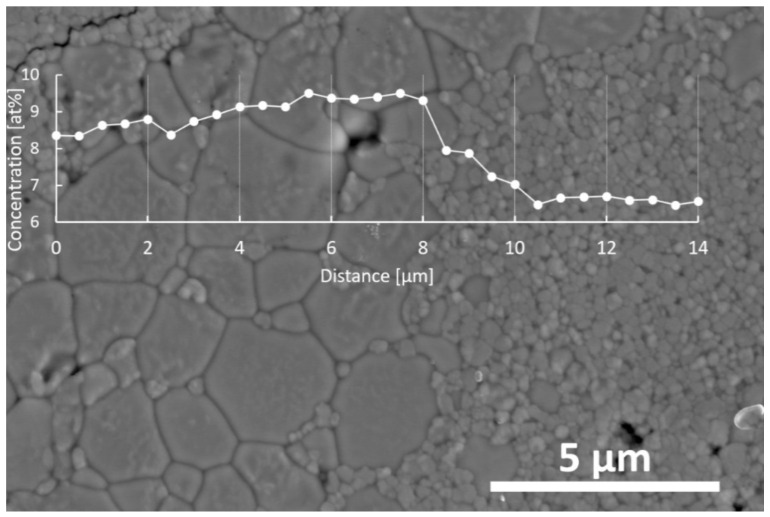
Changes of yttrium concentration across the indicated line of the sample sintered at 1400 °C for 2 h.

Figure 6 shows the microstructure of the sample sintered at 1400 °C for 2 h. We noticed the presence of two grain populations essentially differing in their sizes. It seems reasonable to assume that the larger grains originated from those prepared by the crystallization of the pure zirconia particles in the NaOH solution. However, the line scan EDS analyses of the bigger grains displayed a substantially higher yttrium content than in the much smaller grains surrounding them.

In order to explain the reason for the transfer of yttria from the nanometric yttria–zirconia powders to the pure zirconia grains, two driving forces should be considered. One is obvious and comes from the yttrium concentration gradient. However, the other one should be related to the large curvature at the contact points between the nanometric yttria–zirconia ss particles and the larger grains originating from the pure zirconia grains (Figure 6). We found that the contact boundaries between the larger grains were flat. The latter driving force led to the transfer of the nanometric particle matter toward an order of magnitude initially larger than that of the pure zirconia elongated grains. This is why the yttrium concentration in the larger grains of the system became essentially larger than in the small grains. The mechanism described here operates as long as such contact points exist in the system. That is why the latter mechanism does not allow for the chemical homogenization of the system, at least within the heat treatment conditions applied in this study.

**Figure 7:**
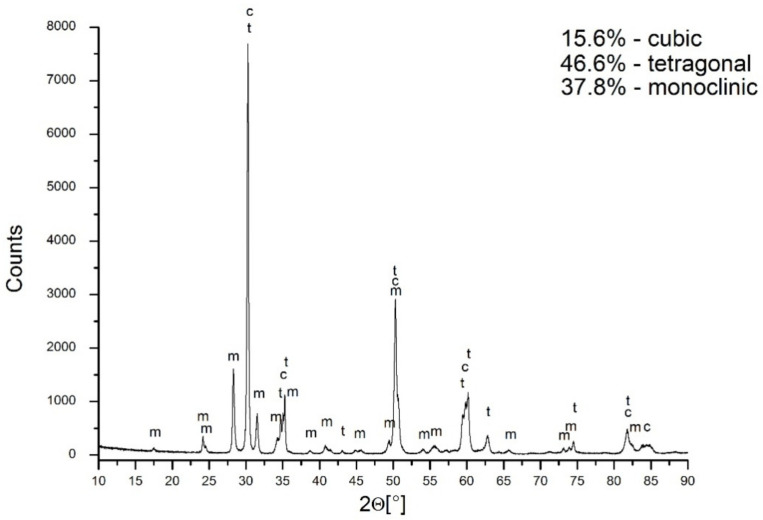
XRD of sintered sample at 1400 °C for 2 h.

The X-ray diffraction of the material under discussion revealed the presence of a 46.6% tetragonal symmetry phase, a 15.6% cubic phase, and a 37.8% monoclinic phase (Figure 7). The problem to be solved is the attribution of symmetry to the grains observed in the sample microstructure. In this case, the application of EBSD proved to be helpful.

In the selected area, the EBSD phase maps showed the presence of two phases: tetragonal (P42/nmc space group) and monoclinic (P21/c space group). The lattice parameters were: a = 0.35958 and nm c = 0.1844 nm for t-YTP, and a = 0.5184 nm and c = 0.53154 nm for m-YSZ.

**Figure 8:**
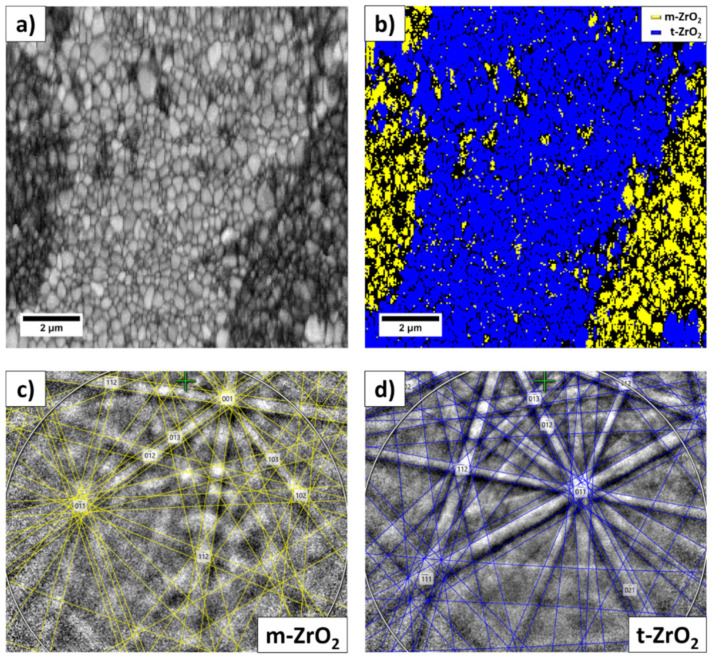
EBSD data of the 3.5 mol% Y_2_O_3_-ZrO_2_ sintered at 1400 °C for 2 h: (**a**) band contrast (pattern quality) map showing basic grain structure, (**b**) phase map, (**c**) indexed Kikuchi patterns of the m-ZrO_2_, and (**d**) indexed Kikuchi patterns of the t-ZrO_2_.

The band contrast (BC) map of the EBSD data performed in the area of interest in Figure 8a confirms that it was fully crystallized and the grain structure appears much more homogeneous than the contrast in the SEM micrograph; all the indexed data points are attributed to the ZrO_2_ phase, with different symmetry. Figure 8b is an EBSD phase map showing the m-ZrO_2_ phase as yellow and the t-ZrO_2_ phase as blue. The fine grain structure was identified as the m-ZrO_2_ phase, which features a monoclinic crystal structure and belongs to the P21/c space group (Figure 8c), while the t-ZrO_2_ phase, characterized by the larger lighter grains shown in Figure 8a, features a tetragonal crystal structure and belongs to the P42/nmc space group (Figure 8d).

In summary, we state that the preferential matter transport from nanometric Y_2_O_3_-ZrO_2_ particles towards sub-micrometer particles leads to the transformation of the latter to form a higher-symmetry part of the system.

Some of the properties of the material sintered at 1400 °C for 2 h are shown in Table 2. Its density corresponded to >95%. The relatively low strengths and fracture toughness of the material most probably resulted from the high content of the monoclinic symmetry phase.

## 4. Discussion

The studied system was composed of nanometric yttria–zirconia solid solution particles and sub-micrometric particles of pure zirconia. Both powders were prepared by the application of crystallization under hydrothermal conditions to the precipitated relative gels. The nanometric particles were crystallized in a water environment, and the sub-micrometic particles were grown in an NaOH aqueous solution.

Good homogenization of a mixture of both powders was achieved by the ultrasonic agitation of the powders’ suspensions, followed by their freeze-drying. These powder compacts indicated a uniform pore size distribution. The sintering of the compacts demonstrated a microstructure material composed of relatively large-micrometer grains and particles one order of magnitude smaller. The former most probably originated from those crystallized in the NaOH aqueous solution and the latter from those crystallized in water.

The EDS analyses showed that large grains contain more yttrium than smaller ones. This means that during heat treatment, the matter of small grains diffuses from particles that are initially nanometric towards particles that are initially sub-micrometric. Two mechanisms could be said to be behind this phenomenon. One results from the yttrium concentration gradient. However, this process should lead to the chemical homogenization of the system. As this is not the case, another driving force leading to the enrichment of larger grains with yttrium should be considered. This is related to the high curvature of the contact points between small and larger grains, which are very visible in the microstructure of the material. This leads to the matter diffusion of smaller grains toward larger ones. These grains, coming initially from sub-micrometric and monoclinic particles, become sufficiently rich in yttrium to develop tetragonal and cubic phase symmetry. Simultaneously, nanometric particles initially rich in yttrium transfer to the part of the microstructure featuring monoclinic symmetry. The EBSD analysis of the selected part of the microstructure illustrates this conclusion. The described phenomenon does not allow for the chemical homogenization of the system, at least within the sintering conditions applied in this study.

## Figures and Tables

**Table 1:** Powder characteristics: specific surface area (Sw), phase composition, and crystallite size (D_hkl_).

Powder	Sw [m^2^/g]	Phase Composition	D_hkl_ [nm]
ZrO_2_ crystallized in NaOH	9.1	Monoclinic	D_002_ = 184 D_020_ = 268 D_200_ = 126
3.5 mol% Y_2_O_3_-ZrO_2_ crystallized in H_2_O	131.5	Tetragonal	D_011_ = 7.5

**Table 2:** Vickers hardness (HV), critical stress intensity factor (K_Ic_), strength (σ), and density (d).

Sintering Conditions	HV [GPa]	K_Ic_ [KPa∙m^1/2^]	σ [ΜPa]	d [g/cm^3^]
1400 °C 2 h	10.3 ± 0.7	5.9 ± 0.9	300 ± 39	5.846 ± 0.003

## Data Availability

The data presented in this study are available upon request from the corresponding authors.

## References

[1] Gupta T.K. (1978). Sintering of tetragonal zirconia and its characteristics. Sci. Sinter..

[2] Gupta T.K., Bechtold J.H., Kuznickie R.C., Cadoff L.H., Rossing B.R. (1977). Stabilization of tetragonal phase in polycrystalline zirconia. J. Mater. Sci..

[3] Gupta T.K., Lange F.F., Bechtold J.H. (1978). Effect of stress induced phase transformation on the properties of polycrystalline zirconia containing metastable tetragonal phase. J. Mater. Sci..

[4] Chaim R., Heuer A.H., Brandon P.G. (1986). Phase equilibration in ZrO_2_-Y_2_-O_3_ Alloys by Liquid Film migration. J. Am. Ceram. Soc..

[5] Pawłowski A., Bućko M.M., Pędzich Z. (2002). Microstructure evolution and electrical properties of yttria and magnesia stabilized zirconia. Mater. Res. Bull..

[6] Pyda W., Haberko K., Bućko M.M. (1991). Hydrothermal Crystallization of Zirconia and Zirconia Solid Solutions. J. Am. Ceram. Soc..

[7] Bućko M.M., Haberko K., Faryna M. (1995). Crystallization of Zirconia under Hydrothermal Conditions. J. Am. Ceram. Soc..

[8] Lach R., Wojciechowski K., Zientara D., Haberko K., Bucko M.M. (2017). Zirconia nano-powder—A useful precursore to dense polycrystals. Ceram. Int..

[9] Niihara K., Morena R., Hasselma D.P.H. (1982). Evaluation of K_Ic_ of brittle solids by the indentation method with low crack-to-indent ratios. J. Mater. Sci. Lett..

[10] Niihara K. (1983). A fracture mechanics analysis of indentation indentation induced Palmqvist crack in ceramics. J. Mater. Sci. Lett..

[11] Scott H.G. (1975). Phase relationship in the zirconia-yttria system. J. Mater. Sci..

